# Testosterone Supplementation Improves Carbohydrate and Lipid Metabolism in Some Older Men with Abdominal Obesity

**DOI:** 10.4172/2167-7182.1000159

**Published:** 2014-06-07

**Authors:** FR Sattler, J He, J Chukwuneke, H Kim, Y Stewart, P Colletti, KE Yarasheski, TA Buchanan

**Affiliations:** 1 Department of Medicine, University of Southern California, Los Angeles, CA, USA; 2 Department of Preventive Medicine, University of Southern California, Los Angeles, CA, USA; 3 Department of Radiology, University of Southern California, Los Angeles, CA, USA; 4 Department of Medicine, Washington University, St. Louis, MO, USA

**Keywords:** Obesity, Insulin resistance, Testosterone, Aging, Metabolism

## Abstract

**Background/Objectives:**

The effects of testosterone supplementation on carbohydrate and lipid metabolism in obese older men are uncertain. We conducted a single-arm open-label prospective pilot study to investigate the effects of testosterone supplementation on central and peripheral insulin sensitivity in older men with upper body obesity and insulin resistance.

**Subjects/Methods:**

Twenty men (62–78 years-old) with morning testosterone levels <13.9 nmol/L (400 ng/dL), waist circumference ≥ 102 cm, and HOMA-IR ≥ 4.0 or HgbA1C 5.7–6.4% applied transdermal testosterone (10 mg) daily for 20 weeks. Insulin sensitivity (Si) was determined by a 2-stage glucose clamp, liver and intramyocellular lipid by ^1^H-MR spectroscopy and body composition by DEXA.

**Results:**

Testosterone supplementation significantly reduced total fat (−.9 ± 2.4 kg, p=0.002), trunk fat (−1.3 ± 1.4 kg, p=0.0007) and extremity fat (−0.7 ± 1.1 kg, p=0.01), and increased extremity lean tissue (+1.3 ± 1.4 kg, p=0.0006). Whole body (WB) Si improved by 21% (0.76 ± 1.57 dL/min per µU/mL, p=0.04) and insulin-stimulated glucose uptake (Rd) by 24% (0.91 ± 1.74 dL/min per µU/mL, p=0.03). Improvements in glucose kinetics were limited to men with reductions in trunk and extremity fat greater than median declines for the entire group. Reductions in intramyocellular lipid were associated with improvements in WB Si (p=0.04) and Rd (p=0.03). Change in Rd accounted for 90% of the change in WB Si. Hepatic glucose output and liver lipid/H_2_O were unchanged (p>0.05). Multivariable analyses revealed that reductions in extremity fat, trunk fat, and FFA levels during the clamp accounted for 45% (p=0.004), 31% (p=0.002) and 8% (p=0.04) of respective changes in Rd. Triglycerides decreased by −0.40 ± 0.67mmol/L (p=0.02), LDL-C by-0.35 ± 0.57 mmol/L (p=0.02), and HDL-C by −0.14 ± 0.19 mmol/L (p=0.004).

**Conclusions:**

Testosterone supplementation that resulted in greater reductions in regional adiposity was associated with improved insulin sensitivity, lower LDL-C and fasting triglycerides, but lower HDL-C. Placebo controlled trials need to further examine the potential cardiometabolic risks/benefits of androgen supplementation for older men with low testosterone levels, central obesity, and insulin resistance.

## Introduction

Guidelines for testosterone supplementation therapy in older men with biochemical and clinical manifestations of hypogonadism are available in the United States and Europe [1,2]. Many of these men also have upper body obesity, as nearly 70% of Americans are obese or overweight [3], the prevalence of pre-diabetes has doubled [4] and these increase with aging. Obesity and its associated insulin resistance are components of the metabolic syndrome [5], which are associated with heart attack, stroke, peripheral vascular disease and amputation, and risk for type 2 diabetes. Obesity and insulin resistance, as well as the full metabolic syndrome, are associated with lower testosereone levels [6–9]. Low testsoterone levels correlate inversely with the magnitude of abdominal obesity [10–12]. Further, hypogonadal men have an increased risk for metabolic syndrome [13]. Thus, it is important to understand how testosterone supplementation when it is administered according to treatment guidelines affects components of the metabolic syndrome in older hyogonadal men with upper body obesity and insulin resistance.

The effects of testosterone administration on upper body obesity have been mixed [14], as have the effects of testosterone treatment on insulin sensitivity [15–17]. Those differences may be related to the dose of testosterone, changes in blood and tissue androgen levels, or duration of therapy [16,18]. In castrated rats, restoration of testosterone to physiologic levels improves insulin sensitivity, but supraphysiologic levels worsen insulin resistance [19]. Therefore, we administered transdermal testosterone to older men with low morning testosterone levels and components of the metabolic syndrome, and investigated the effects of treatment on upper body obesity, insulin resistance and serum lipids. We determined which participants were most likely to respond favorably to the intervention and whether the effects were largely central (affecting hepatic glucose output and hepatic lipid content) or peripheral (affecting glucose disposal and intramyocellular lipid content [IMCL]). We postulated that men with large declines in trunk or extremity fat would have the greatest improvements in insulin sensitivity. To achieve these aims, men were phenotyped in a clinical trials research unit using a two-stage hyper insulinemiceu glycemic clamp and proton magnetic resonance spectroscopy.

## Subjects and Methods

### Study design

Older men with low morning total testosterone levels typical of their age, upper body obesity, and evidence of insulin resistance applied 10g of 1% transdermal testosterone gel (Androgel, Solvay Pharmaceuticals Inc.) daily for 20 weeks (open label). This was a pilot project that utilized complex and intensive research measurements, so that each subject served as his own control with the expectation that outcomes would be sizable and beyond what would occur by chance. Three-day food diaries and exercise questionnaires were collected at baseline, week 10 and week 20 to confirm that subjects did not change their total energy or macronutrient intake and activity levels by more than 10% during the study. Blood tests for complete cell counts, chemistries, prostate specific antigen (PSA), and testosterone were done at baseline, weeks 10 and 20 and 3-months after completion of study therapy to monitor safety.

### Study participants

Participants provided written informed consent approved by the USC Institutional Review Board. Eligible men were 60 years or older, with morning total testosterone concentrations <13.9 nmol/L (400 ng/dL) typical of older men with low levels and often with symptoms of hyopgonadism, waist circumference ≥ 102 cm (average of 3 measurements by a bionutritionist), and evidence consistent with insulin resistance; HgbA1c of 5.7–6.4% [20] or HOMA-IR ≥ 4.0, based on values above the 50th percentile in our prior studies of obese men ≥ 60 years old. Exclusion criteria included fasting plasma glucose ≥ 6.93 mmol/L (126 mg/dL) or history of diabetes, PSA ≥ 4.0 µg/L, hematocrit ≥ 50%, AST ≥ 2X the upper-limit-of-normal, clinical evidence of a concurrent inflammatory process (rheumatoid arthritis, active infection, etc), vigorous exercise >30 min/week (existing stable walking programs were allowed) or plans to reduce energy intake.

### Two stage hyperinsulinemic euglycemic clamp

#### Clamp procedure

Two stage hyperinsulinemic-euglycemic clamp studies were conducted to determine whole body (WB) insulin sensitivity (Si) and effects of insulin on the rates of peripheral glucose disposal (Rd) and hepatic glucose output (HGO) [21]. Briefly, regular insulin was infused continuously in a right forearm vein at 4 ml/hr (0.3 mUnits/kg/min) during minutes 0–120; this rate was increased to 13.3 ml/hr (1.0 mUnits/kg/min) during minutes 120–240. During the clamp, blood glucose was maintained at 4.72–5.00 mmol/L (85–90 mg/dL) by a variable infusion of 20% dextrose, based on bedside glucose levels (YSI analyzer) determined every 5 min using specimens collected from a dorsal left hand vein heated by a Gaymar T-pump (95–100°F). At −30, −20, −10, +100, +110, +120 and +220, +230, and +240 minutes, samples were collected from the left hand vein, processed immediately for plasma and stored at −80°C for batch analysis.

#### Calculation of insulin sensitivity

Clamp insulin sensitivity index (Si) was determined as reported previously [21]. Whole-body (WB) Si was calculated as ΔGinf / (ΔIns X Gluss), where ΔGinf and ΔIns are the respective increments in the dextrose infusion rate and plasma insulin concentrations between basal (average at −30, −20, −10 min) and steady state (average of +220, +230, +240 min); Gluss is the steady-state glucose concentration. Peripheral insulin sensitivity was determined by the rate of glucose disposal (Rd); calculated as ΔRd / (ΔIns X Gluss), during hours three and four of the clamp. Hepatic insulin sensitivity was calculated to determine the effects of insulin on hepatic glucose output (HGO), where Si HGO = ΔHGO / (ΔIns X Gluss), from basal to steady state at the end of hour 2.

#### Hormone Assays

Screening testosterone levels were quantified by liquid chromatography-tandem mass spectrometry (Quest Diagnostics, San Juan Capistrano). Samples for insulin, glucose, and free fatty acids (FFAs) collected during the glucose clamps were batch tested at the USC Clinical Trials Unit (CTU) Core Laboratory. Plasma glucose levels were determined using an YSI glucose analyzer (Yellow Springs, OH) with a coefficient of variation (CV) = 2% and detection range=0–50.0 mmol/L (0–900 mg/dL). Insulin levels were determined by an automated enzyme immunoassay (Tosoh AIA 600 II analyzer, Tosoh Bioscience, Inc., South San Francisco, CA; sensitivity=0.31 µIU/mL, inter-assay CV=6.1% and intra-assay CV=4.8%). HOMA-IR was calculated as fasting insulin (µU/ml) X fasting glucose (mM)/22.5. FFA levels were determined in duplicate at the CTU Core Laboratory by an automated immunoassay method (Wako Diagnostics, Richmond, Virginia); inter- and intra-assay CVs were 0.75% with a sensitivity of 0.0014 mEq/L, respectively.

### Body composition

#### Dual Energy X-ray Absorptiometry (DEXA)

Total, trunk, and appendicular lean tissue and fat mass were quantified using a Hologic QDR 4500 Discovery Scanner (Holoigic Inc, Bedford, MA). Pre and post-treatment scans were blindly analyzed by a single DEXA-certified technician using software pre-installed on the machine.

#### Proton magnetic resonance spectroscopy

Proton (^1^H) MR spectroscopy was performed with either 1.5T or 3T General Electric scanners. For evaluation of the liver fat fraction, the proton signal was obtained using the torso coil from the 8cm^3^ PRESS (point resolved spectroscopy) voxel in the right posterior superior segment of the liver (32 average, 35 ms echo time, 2s repetition time). For assessment of the intramyocellular lipid (IMCL), the proton signal was obtained using a knee coil from the voxel of 1.00-to-2.25 cm^3^ prescribed on the T1 and T2 -weighted images of the anterior tibialis. The PRESS voxel was positioned where axial slices of the superior aspect of the anterior tibialis are large enough to avoid contribution from subcutaneous fat and visible fatty layers between adjacent muscles [22]. Each spectrum was averaged by 32 or 64 acquisitions with TE=35 ms, TR=2s. Proton metabolites were quantified using AMARES algorithm in the jMRUI [23]. The fat peak, 3.5 ppm apart from the water, was normalized to the water peak for the evaluation of liver fat/water fraction. IMCL was evaluated with an 8-peak lipid model, where the methylene peak of the IMCL resonances, (CH_2_)N, was identified at approximately 1.3 ppm normalized to the creatine peak (3.02 ppm) [24]. Hepatic lipid/H_2_O ratios were obtained on 19/20 participants. In 4 of these participants, intra- and extra-myocellular lipids could not be clearly separated in baseline and week 20 spectra; thus, 15 participants had good acquisition pairs for IMCL/Cr analyses.

### Statistical considerations

#### Baseline characteristics

Baseline characteristics are reported for the entire cohort and for two subgroups based on reductions in trunk fat mass above or below the median change at week 20. Continuous variables were compared using Wilcoxon rank-sum test between the two subgroups. Categorical variables were examined by the Fisher’s exact test due to the small sample size.

#### Change in study outcomes

Paired t-tests were used to compare baseline values to the week 20 outcomes of serum testosterone levels, body composition, insulin sensitivity, and serum lipids. Associations between Si clamp values and other potential predictors were analyzed by Spearman’s correlation. Predictors of Si WB and Si Rd were identified through regression analyses. Their relative contributions were quantified by multivariable analyses to determine partial R^2^. To further examine the effects of fat reduction on the changes in Si clamp values, the cohort was divided into those with high and low changes (above and below the median changes) in trunk and extremity fat. This analysis was based on our a priori postulate that men with reductions in trunk fat >1.0 kg or extremity fat >0.7 kg would have greater changes in Si variables. Two-sample t-tests were used to compare the changes of all three Si clamp measures between participants in the high and low fat change groups.

An alpha level of p ≤ 0.05 was considered statistically significant. Means ± one SD and medians with range values are reported. Statistical analyses were carried out using Statistical Analysis System 9.2 (Cary, NC).

#### Sample size and power calculations

For this pilot project to generate preliminary data, we chose N=20 participants. We determined that this sample size would provide 80–90 % statistical power to show pre-to-post changes in the primary outcomes for effect sizes of 0.5–0.7 with p<0.05.

## Results

At baseline, the participants were representative of older men with upper body obesity (Table 1). After testosterone supplementation, the median change in trunk fat was −0.86 kg; 10 men lost ~1.0 kg or more, and 10 men lost <0.8 kg. Baseline characteristics for the two groups were generally similar, but the group with larger reductions in trunk fat had higher baseline HOMA-IR, an index of insulin resistance. Morning serum testosterone levels increased from baseline (10.1 ± 2.78 nmol/L; 292 ± 80 ng/dL) to week 20 (21.5 ± 11.1 nmol/L; 619 ± 320 ng/dL; p=0.0002). Week 20 testosterone levels are typical of those for young men.

### Changes in insulin sensitivity index

During the glucose clamp, whole body (WB) Si increased 0.76 ± 1.57dL/min per µU/mL (p=0.04) and Rd increased 0.91 ± 1.74 dL/min per µU/mL (p=0.03, Table 2). However, HGO did not change significantly. HOMA-IR declined marginally (p=0.06).

### Fasting lipids and free fatty acid levels

Significant reductions in fasting triglycerides, total cholesterol, and LDL-C (p ≤ 0.02, Table 2) levels occurred, but HDL-C also declined, albeit modestly by 0.14 ± 0.19 mmol/L (−5.3 ± 7.2 mg/dL; p=0.004). Basal free fatty acid levels (FFA) did not change over the 20 week treatment period (Table 2). Levels of FFA did correlate negatively with baseline WB Si, HGO, and Rd (ρ= −0.63, −0.58, −0.59, respectively, p ≤ 0.02 for each).

### Relationship of change between Si and other parameters

Changes in total, trunk and extremity fat mass as well as percentage of total fat correlated with changes in WB Si (rho = −0.45 to −0.52; p ≤ 0.05) and for Rd (rho = −0.46 to −0.59, p ≤ 0.02) but not with HGO (data not shown). Changes in lean tissue mass and hepatic lipid were not related to any Si measure. In 12 of 15 participants, IMCL/Cr declined (mean = −41%) and corresponded with mean increases in WB Si of 0.82 (p=0.06) and Rd of 0.93 dL/min per µU/mL (p=0.04).

### Factors associated with change in whole body Si and Rd

**Figure 1:**
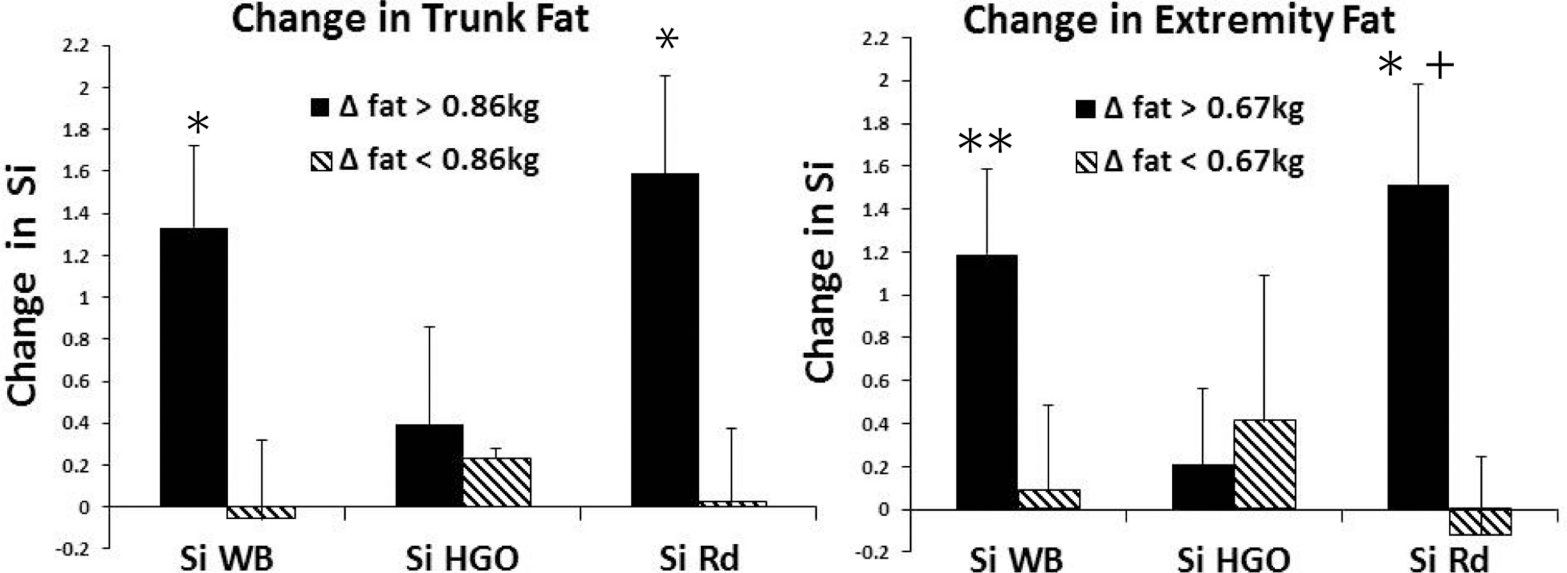
Changes in Si by Change in Trunk and Extremity Fat Mass. Median change in trunk fat = −0.86 kg and for extremity fat = −0.67 kg. Change in Si is in units of dL/min per µU/mL. *within group change p ≤ 0.05; ** within group change p = 0.06; † between group change p = 0.09

WB Si and Rd each improved significantly for men with reductions in trunk and extremity fat greater than the respective median changes (Figure 1). The effects of change in total fat on WB Si and Rd were largely due to the summation of changes in trunk plus extremity fat since together they consisted of almost 100% of the changes in total fat (data not shown). Univariable analyses were conducted to assess potential contributors to change in WB Si that included baseline HOMA-IR, Si WB, and fat mass (total and regional) as well as changes in Si Rd, Si HGO, total and regional fat and lean tissue, IMCL/Cr, hepatic lipid/H_2_O, FFAs, and fasting lipids. Those with p ≤ 0.30 were included in multivariable models. Two models best described change in WB Si (Table 3). In one model, Rd accounted for 90% (p<0.0001) and Si HGO accounted for 9% (p<0.0001) of the change in WB Si. If only Rd and HDL-C were in the model, Rd still accounted for 90% (p<0.0001) and HDL-C 3% (p=0.01) of the change in WB Si. Thus, Rd accounted for most of the improvements in WB Si. To evaluate contributing factors for Rd, we used the same variables for univariable analyses to investigate potential contributors to change in WB Si. The multivariable model that included change in extremity fat, trunk fat, and FFAs, accounted for 45% (p=0.004), 31% (p=0.002) and 8% (p=0.04) of the respective change in Rd (Table 3).

### Changes in body composition

After 20 weeks of testosterone replacement average total body mass was unchanged (Table 2). Total, trunk and extremity fat mass declined 1.9 ± 2.4 kg (maximum=7.4 kg), 1.3 ± 1.4 kg (maximum=4.6 kg) and 0.7 ± 1.1 kg (maximum=3.0 kg), respectively (p ≤ 0.01 for each). IMCL/Cr ratios decreased 28 ± 35% (maximum=85%; p=0.008) but hepatic lipid/H_2_O was unchanged. Total LBM increased 2.1 ± 2.0 kg (maximum=7.1 kg, p=0.0002) as did extremity LBM by 1.3 ± 1.4 kg (maximum=4.8 kg, p=0.0006).

### Adverse effects from testosterone therapy

Treatment was generally well-tolerated and there were no serious adverse events. Hematocrit and PSA did not increase significantly whereas American Urological Association urinary obstruction scores declined (ANOVA p=0.04). Framingham 10-year cardiovascular risk scores at baseline (18.7 ± 6.01%) remained similar over the ensuing 32 weeks (ANOVA p=0.90).

## Discussion

In this mechanistic 20 week pilot study, transdermal testosterone (10 g of a 1% gel daily) supplementation in older obese men achieved serum testosterone levels typical of men in their 3rd and 4^th^ decades of life. This regimen reduced total fat mass by an average of 1.9 kg and reciprocally increased total lean mass by 2.1 kg, similar to changes reported in treatment studies [25,26].

Insulin sensitivity for whole body and peripheral glucose disposal improved significantly, but only in men that lost more than the median reduction of adipose tissue (>0.86 kg trunk fat or >0.67 kg extremity fat). Total cholesterol, LDL-C and fasting triglycerides decreased across the study population, although HDL-C declined modestly by 0.1 mmol/l (3.9 mg/dL). Decreases in HDL-C or adiponectin as reported could be associated with increases in inflammation [27]. Yet, on balance testosterone supplementation had favorable effects on three of the major components of the metabolic syndrome including LDL-C and triglycerides in most of the older men.

A primary goal was to identify the principal target tissue (central versus peripheral) of the insulin sensitizing actions of testosterone in these older, centrally obese men. Improvements in whole body Si were driven largely by enhanced peripheral glucose disposal (~90%), and 45% of the improvements in Rd were associated with reductions in extremity fat mass. This suggests that testosterone primarily affects peripheral insulin sensitivity. Improvements in whole body Si and Rd were not related to enhancements in muscle mass despite increases in extremity lean mass (~1.2 kg), which is largely muscle. However, testosterone treatment was associated with reductions in IMCL. This is consistent with reports indicating that IMCL is more tightly associated with insulin resistance than BMI, waist-to-hip ratio or total body fat [28,29]. Elevated IMCL probably reflects accumulation of long chain acyl-CoA. This can activate protein kinase C, lead to serine (rather than tyrosine) phosphorylation on insulin response substrate-1 (IRS-1), and limit PI-3 kinase activation which is necessary for translocation of GLUT4 to the cell membrane for intracellular transport of glucose [30]. Thus, reducing IMCL should improve muscle glucose disposal. Further, in vitro data indicate that testosterone directly up-regulates IRS-1 signaling and GLUT4 transporter activity, as expected to occur in muscle. Finally, testosterone increases mitochondrial function and oxidative phosphorylation capacity in muscle, which may result in improved insulin sensitivity [31,32]. Thus, testosterone may regulate muscle metabolism by several different mechanisms that improve whole body insulin sensitivity and the peripheral rate of glucose disposal.

Nevertheless, it is possible that testosterone affects aspects of central metabolism that could improve whole body Si despite lack of improvement in hepatic glucose output as occurred in our participants. Testosterone is expected to reduce visceral adipose tissue (VAT, namely omental and mesenteric fat) and prevents its increase during aging [33]. VAT is more sensitive to lipolytic stimuli than subcutaneous fat [33,34], which is expected to reduce FFA release to the liver through the portal circulation. Although we did not quantify changes in VAT and SAT, we expect that a major portion of the reductions in trunk fat by DEXA were due to decreases in VAT, despite evidence that treatment in some aging men may be limited to significant reductions in subcutaneous abdominal or extremity fat [35]. Regardless, hepatic lipid content by proton MRS was not changed suggesting that any reductions in VAT were not associated with significant decreases in FFA transport through the portal circulation that would increase hepatic gluconeogenesis [36]. This is consistent with our observation that insulin-mediated suppression of HGO, a measure of hepatic insulin sensitivity, was not affected by treatment with testosterone. Testosterone increases lipolysis globally [37,38], which should ultimately lower systemic FFA levels. In our study, reductions in FFA during the clamp after 20 weeks of testosterone treatment accounted for 8% of the improvement in Rd. Finally, acute changes in testosterone may modulate insulin sensitivity even without changes in body fat [8,9,19].

Total body mass did not change due to off-setting increases in lean mass and reductions in fat mass. At least in vitro, testosterone can direct pluripotent stem cells down a myogenic lineage, inhibit adipogenesis [39], and inhibit differentiation of preadipocytes into mature fat cells; this occurs by inhibition of β-catenin nuclear translocation and its downstream effects on Wnt signaling necessary for differentiation of adipocytes [40]. These mechanisms support our observed in vivo actions of testosterone--comparable alterations in lean versus fat mass as reported by others [18,41]. Taken together, it appears that testosterone supplementation improved peripheral insulin sensitivity through multiple biochemical and cell-signaling pathways that regulate muscle and adipose tissue metabolism.

The goal of this study was not to investigate the effects of testosterone for treatment of Metabolic Syndrome but to determine the effects of treatment, when used according to published guidelines, on components of the Syndrome in older men with upper body obesity. In these men, baseline morning testosterone levels were 13.9 nmol/L (<400 ng/dL) which is higher than the commonly used breakpoint for treatment of symptomatic hypogonadism (10.4 nmol/L [<300 ng/dL]). This threshold was determined with older platform immunoassays that were regularly used when treatment guidelines were established. However, with newer analytical platforms, like LC-MS/MS used in this study, the normal reference range is >12.08 nmol/L (>349 ng/dL) [42], and using GC-MS, the threshold at which symptoms associated with low testosterone are more common is <11 nmol/L (<320 ng/dL) [43]. Most participants in our study had levels below these thresholds and, thus, were generally similar to many men who would be candidates to receive clinically indicated testosterone replacement, if they are symptomatic.

The treatment dose and duration achieved levels typical of young men [of at least 20.8 nmol/L (600 ng/dL)] in 17 (85%) participants and provided improvements in components of the Metabolic Syndrome. Further, there were no important adverse events and the 10 year Framingham cardiovascular disease risk scores, which were just under the high risk category of 20%, remained unchanged after 20 weeks of testosterone replacement and for the next three months after treatment was discontinued.

This study is unique with respect to phenotyping methods (glycemic clamp, IMCL, hepatic lipid) and the eligibility criteria: older men with abdominal obesity and components of the Metabolic Syndrome. Four studies of testosterone supplementation in older men found no beneficial effects on surrogate measures of Si, but none used the glycemic clamp and older men were not selected for upper body obesity [44–47]. Whereas, two studies have shown improvements in Si by glycemic clamps in obese middle aged men (younger than our men) [15,48]. One recent 6-month study using a different 50–100 mg testosterone gel in 62–72 year-old-men with “obesity” showed no improvement in Rd versus placebo [35]. However, some of their men did not have upper body obesity (waist circumference 94–101 centimeters) by metabolic syndrome criteria. Importantly, trunk and lower extremity fat did not decrease significantly, which may explain the lack of improvement in Rd [49]. Whereas, in our study significant improvements in WB Si and Rd occurred only in centrally obese men who had sizeable reductions in trunk, extremity, and intramuscular fat content. Thus, relatively large reductions in these fat compartments may be needed to achieve improvements in insulin sensitivity in older men with upper body obesity and components of the Metabolic Syndrome.

There were several limitations. First, the number of participants (N=20) was relatively small and there was no control group. The significant reciprocal increases in lean tissue, including extremity muscle mass, and significant decreases in total and trunk fat would not have occurred with diet alone or with aerobic exercise, and none of our participants were doing resistance exercise to increase lean mass. Three-day food diaries and exercise questionnaires (data not shown) did not change during or for the three months after therapy. Thus, changes in body composition that affected Si can be reasonably attributed to treatment with testosterone. Second, we did not quantify changes in abdominal fat compartments (VAT and SAT), which presumably decreased based on reductions in trunk fat by DEXA. We recognize that reductions in VAT may decrease release of proinflammatory cytokines, which should facilitate pathways enhancing insulin sensitivity. Finally, our study was relatively short with treatment lasting only 20 weeks. We can only conjecture as to whether longer therapy (e.g. 1–3 years) would result in greater reductions in fat mass and IMCL and whether this would be associated with even more favorable effects on clinically important measures of metabolism. Regardless, we believe that our results using the two stage glucose clamp for HGO and Rd along with MRS for quantifying hepatic lipid and IMCL provide compelling direct and inferential evidence that testosterone improved insulin sensitivity by largely peripheral effects, but changes in VAT per se and IL-6 and TNFα levels should be quantified in future studies.

The long term safety of testosterone replacement remains uncertain as adverse cardiovascular events have been reported in some high risk populations [50–53]. Results of this study are not intended to support or promote use of testosterone for treatment of aging or other perceived benefits but to understand the cardiometabolic effects of testosterone if it is prescribed according to standardized guidelines. Further, the long term safety of testosterone supplementation for low levels in symptomatic persons has not been ascertained [54–56]. Thus, before prescribing testosterone for symptoms of hypogonadism in older men, the full spectrum of potential risks and benefits should be considered for each patient. As part of these considerations, our results suggest that treatment may beneficially affect important components of metabolism in some patients, especially in those with sizable amounts of upper body fat mass. Based on results of our prior studies and those of others [18,25,26], it is unclear why testosterone supplementation produces larger reductions in fat mass up to 6–7 kg in some men with very little change in other men. Regardless, larger, placebo controlled studies are needed to confirm these promising preliminary findings and the long term safety of testosterone supplementation. Finally, it will be important to determine why certain men may have substantial reductions in upper body fat and muscle lipid along with improvements in insulin sensitivity and serum lipids, while these are not affected in other obese older men receiving testosterone.

## Figures and Tables

**Table 1:** Study Participant Characteristics

	Total Subjects	Δ inTrunk fat >0.86 kg a	Δ inTrunk fat <0.86 kg	P value
Characteristics	n = 20	n = 10	n = 10	
Age,*years*	67.5 (62.0, 78.0) b	67.0 (62.0, 78.0)	68.0 (63.0, 77.0)	0.65
Weight, *kg*	105 (85,137)	113 (89, 137)	100 (85, 127)	0.09
Family history of diabetes	4 (20%)	1 (10%)	3 (10%)	0.58
Smoking ever/currently	14 (70%)/0 (0%)	9 (90%)	5 (50%)	0.14
On treatment for lipid disorder	12 (60%)	7 (70%)	5 (50%)	0.65
On treatment for hypertension	10 (50%)	6 (60%)	4 (40%)	0.66
BMI, *kg/m*^2^	32.0 (27.3, 41.7)	34.8 (29.7, 41.7)	30.6 (27.3, 39.9)	0.12
Waist circumference, *cm*	114 (104, 122)	117 (104, 122)	109 (104, 121)	0.08
Systolic pressure, *mm Hg*	130 (110, 173)	136 (115, 156)	129 (110, 173)	0.45
Fasting glucose, *mmol/L*^c^	5.86 (4.94, 6.66)	6.24 (4.94, 6.55)	5.83 (5.05, 6.66)	0.35
Fasting triglycerides, *mmol/L*^d^	1.59 (0.72, 4.87)	1.60 (0.92, 4.87)	1.52 (0.72, 2.45)	0.85
Total cholesterol, *mmol/L*^e^	4.61 (3.00, 6.73)	4.92 (3.47, 5.57)	4.40 (3.00, 6.73)	0.68
LDL-cholesterol, *mmol/L*^e^	3.11 (0.98, 5.00)	3.13 (1.61, 3.76)	2.73 (0.98, 5.00)	0.90
HDL-cholesterol, *mmol/L*^e^	1.06 (0.62, 1.76)	1.09 (0.78, 1.76)	0.98 (0.62, 1.42)	0.47
HgbA1C, %	5.9 (5.3, 6.3)	6.0 (5.3, 6.3)	5.9 (5.5, 6.1)	>0.99
HOMA-IR	4.81(1.44, 9.32)	6.47 (4.35, 9.32)	4.13 (1.44, 8.06)	0.009
AM total testosterone, *nmol/L*^f^	10.6 (5.2, 13.9)	9.6 (5.2, 13.4)	11.0 (5.9, 13.9)	0.57

a Median change in trunk fat = −0.86 kg;

b median (minimum, maximum);

c for mg/dL, divide by 0.0555;

d for mg/dL, divide by 0.0113;

e for mg/dL, divide by 0.0259;

f for ng/dL, divide by 0.0347

**Table 2:** Change in Cardiometabolic Variables during Study Treatment

Variable	Baseline	Change at Week 20	P value
Body Composition			
Total body mass, *kg*	104.5 (84.4, 133.6) a	0.3 (−4.8, 4.1)	0.77
Total fat mass, *kg*	34.6 (24.3, 57.1)	−1.4 (−7.4, 2.0)	0.003
Trunk fat mass,*kg*	19.3 (13.6, 30.0)	−0.9 (−4.6, 0.2)	0.0007
Extremity fat mass,*kg*	12.9 (9.1, 27.6)	−0.7 (−3.0, 2.3)	0.01
% total body fat	31.5 (23.9, 42.7)	−1.6 (−6.5, 0.1)	0.0002
Hepatic Lipid/H_2_O (n=19) b	0.11 (0.01, 1.13)	−36% (−84,127)	0.12
IMCL/Cr (n=15) c	5.8 (0.2, 30)	−30% (−85, 43)	0.008
Extremity lean mass, *k*^g^	29.1 (21.2, 34.1)	1.2 (−1.1, 4.8)	0.0006
Total lean body mass, *kg*	64.3 (47.1, 75.0)	1.4 (−0.8, 7.1)	0.0002
Insulin Sensitivity			
Whole body (WB) d	2.93 (1.07, 8.51)	1.05 (−2.44, 3.73)	0.04
Hepatic glucose output (HGO) d	2.89 (0.03, 7.83)	−0.16 (−2.58, 7.49)	0.59
Rate of disposal (Rd) d	2.93 (1.07, 8.51)	1.16 (−3.08, 3.85)	0.03
HOMA-IR	3.55 (0.64, 11.4)	−0.75 (−6.94, 4.18)	0.06
Plasma Lipids			
Fasting triglycerides,*mmol/L*^e^	1.59 (0.72, 4.87)	−0.33 (−2.70, 0.43)	0.02
Total cholesterol, *mmol/L*^f^	4.61 (3.00, 6.73)	−0.52 (−1.74, 0.88)	0.004
LDL-cholesterol, *mmol/L*^f^	3.11 (0.98, 5.00)	−0.23 (−1.45, 0.70)	0.02
HDL-cholesterol, *mmol/L*^f^	1.06 (0.62, 1.76)	−0.10 (−0.60, 0.05)	0.004
Basal free fatty acids, *mEq/*^L^	0.35 (0.24, 0.53)	−0.01 (−0.22, 0.21)	0.97
Δ in Clamp FFA g (0–4 hours)	0.30 (0.17, 0.41)	−0.01 (−0.12, 0.17)	0.83

a Median (minimum, maximum).

b Only 19 had proton spectrometry at baseline and week 20.

c In four participants, IMCL and EMCL could not be clearly separated at either baseline or week 20.

d unitsare dL/min per µU/mL;

e for mg/dL, divide by 0.0113;

f for mg/dL, divide by 0.0259;

g free fatty acids

**Table 3:** Predictors of Change in Whole Body Si and Rd

Change (Δ) in Si for Whole Body						Δ in Si for Rd		
Univariable Analysis^a^		Multivariable Analysis 1		Multivariable Analysis 2		Multivariable Analysis	
Variables^b^,^c^	R^2^ (p-value)	Variable^c^,^d^	R^2^ (p-value)	Variable^c^,^e^	R^2^ (p-value)	Variable^c^,^f^	R^2^ (p-value)
Δ Si Rd	90% (0.002)	Δ Si Rd	90% (<0.0001)	Δ Si Rd	90% (<0.0001)	Δ Extremity Fat	45% (0.004)
Δ Si HGO	42% (<0.0001)	Δ Si HGO	9% (<0.0001)	Δ HDL-C	3% (0.01)	Δ Trunk Fat	31% (0.002)
Δ Total Fat Mass	7%(0.25)					Δ FFA 4h–basal	8% (0.04)
Δ Extremity Fat	6%(0.27)						
Δ Triglycerides	8%(0.24)						
Δ HDL-cholesterol	9%(0.21)						

a Other variables included baseline total and regional lean and fat mass, HOMA-IR, and WB Si, and change in trunk fat, total lean body mass (LBM), extremity LBM, hepatic lipid by magnetic resonance spectroscopy (MRS), intramyocellular lipid (IMCL) by MRS, free fatty acids (4hr minus basal), and LDL-cholesterol.

b Variables with p≤0.3 from univariable regression analysis are shown.

c All variables are change from baseline to week 20.

d Model contains only change in Si for Rd and HGO. Other variables were not significant.

e Model contains only change Si Rd and HDL-C. Other variables were not significant.

f Model contains only change extremity fat, trunk fat, and free fatty acids at 4hours minus basal in the clamp. Other variables were not significant

## Abbreviations

AST: Aspartate aminotransferase
DEXA: Dual energy X-ray absorptiometry
ΔGinf: Increments in dextrose infusion rate during the glucose clamp
ΔIns: Increments in plasma insulin during the glucose clamp
FFA: Free fatty acids
Glu-ss: steady-state glucose concentration
HDL: high density lipoprotein
HGO: hepatic glucose output
HOMA IR: homeostatic method of anlaysis Insulin Resistance
IMCL: Intramyocellular Lipid
LDL: Low Density Lipoprotein
MR: Magnetic Resonance
PSA: Prostate Specific Antigen
Rd: Rate of Disposal
Si: Insulin Senstivitiy
SD: Standard deviation
WB: Whole body

## References

[1] Bhasin S, Cunningham GR, Hayes FJ, Matsumoto AM, Snyder PJ (2010). Testosterone therapy in men with androgen deficiency syndromes: an Endocrine Society clinical practice guideline. J Clin Endocrinol Metab.

[2] Nieschlag E, Swerdloff R, Behre HM, Gooren LJ, Kaufman JM (2005). Investigation, treatment and monitoring of late-onset hypogonadism in males: ISA, ISSAM, and EAU recommendations. Int J Androl.

[3] Flegal KM, Carroll MD, Kit BK, Ogden CL (2012). Prevalence of obesity and trends in the distribution of body mass index among US adults, 1999–2010. JAMA.

[4] Selvin E, Parrinello CM, Sacks DB, Coresh J (2014). Trends in prevalence and control of diabetes in the United States, 1988–1994 and 1999–2010. Ann Intern Med.

[5] Grundy SM, Cleeman JI, Daniels SR, Donato KA, Eckel RH (2005). Diagnosis and management of the metabolic syndrome: an American Heart Association/National Heart, Lung, and Blood Institute Scientific Statement. Circulation.

[6] Kupelian V, Page ST, Araujo AB, Travison TG, Bremner WJ (2006). Low sex hormone-binding globulin, total testosterone, and symptomatic androgen deficiency are associated with development of the metabolic syndrome in nonobese men. J Clin Endocrinol Metab.

[7] Laaksonen DE, Niskanen L, Punnonen K, Nyyssönen K, Tuomainen TP (2005). The metabolic syndrome and smoking in relation to hypogonadism in middle-aged men: a prospective cohort study. J Clin Endocrinol Metab.

[8] Pitteloud N, Hardin M, Dwyer AA, Valassi E, Yialamas M (2005). Increasing insulin resistance is associated with a decrease in Leydig cell testosterone secretion in men. J Clin Endocrinol Metab.

[9] Yialamas MA, Dwyer AA, Hanley E, Lee H, Pitteloud N (2007). Acute sex steroid withdrawal reduces insulin sensitivity in healthy men with idiopathic hypogonadotropichypogonadism. J Clin Endocrinol Metab.

[10] Nielsen TL, Hagen C, Wraae K, Brixen K, Petersen PH (2007). Visceral and subcutaneous adipose tissue assessed by magnetic resonance imaging in relation to circulating androgens, sex hormone-binding globulin, and luteinizing hormone in young men. J Clin Endocrinol Metab.

[11] Giagulli VA, Kaufman JM, Vermeulen A (1994). Pathogenesis of the decreased androgen levels in obese men. J Clin Endocrinol Metab.

[12] Zumoff B, Strain GW, Miller LK, Rosner W, Senie R (1990). Plasma free and non-sex-hormone-binding-globulin-bound testosterone are decreased in obese men in proportion to their degree of obesity. J Clin Endocrinol Metab.

[13] Laaksonen DE, Niskanen L, Punnonen K, Nyyssönen K, Tuomainen TP (2004). Testosterone and sex hormone-binding globulin predict the metabolic syndrome and diabetes in middle-aged men. Diabetes Care.

[14] Bhasin S (2003). Effects of testosterone administration on fat distribution, insulin sensitivity, and atherosclerosis progression. Clin Infect Dis.

[15] Mårin P, Holmäng S, Jönsson L, Sjöström L, Kvist H (1992). The effects of testosterone treatment on body composition and metabolism in middle-aged obese men. Int J Obes Relat Metab Disord.

[16] Singh AB, Hsia S, Alaupovic P, Sinha-Hikim I, Woodhouse L (2002). The effects of varying doses of T on insulin sensitivity, plasma lipids, apolipoproteins, and C-reactive protein in healthy young men. J Clin Endocrinol Metab.

[17] Liu PY, Wishart SM, Celermajer DS, Jimenez M, Pierro ID (2003). Do reproductive hormones modify insulin sensitivity and metabolism in older men? A randomized, placebo-controlled clinical trial of recombinant human chorionic gonadotropin. Eur J Endocrinol.

[18] Bhasin S, Woodhouse L, Casaburi R, Singh AB, Mac RP (2005). Older men are as responsive as young men to the anabolic effects of graded doses of testosterone on the skeletal muscle. J Clin Endocrinol Metab.

[19] Holmäng A, Björntorp P (1992). The effects of testosterone on insulin sensitivity in male rats. Acta Physiol Scand.

[20] American Diabetes Association (2012). Diagnosis and classification of diabetes mellitus. Diabetes Care.

[21] Ader M, Kim SP, Catalano KJ, Ionut V, Hucking K (2005). Metabolic dysregulation with atypical antipsychotics occurs in the absence of underlying disease: a placebo-controlled study of olanzapine and risperidone in dogs. Diabetes.

[22] White LJ, Ferguson MA, McCoy SC, Kim H (2003). Intramyocellular lipid changes in men and women during aerobic exercise: a (1)H-magnetic resonance spectroscopy study. J Clin Endocrinol Metab.

[23] Naressi A, Couturier C, Castang I, de Beer R, Graveron-Demilly D (2001). Java-based graphical user interface for MRUI, a software package for quantitation of in vivo/medical magnetic resonance spectroscopy signals. Comput Biol Med.

[24] Boesch C, Machann J, Vermathen P, Schick F (2006). Role of proton MR for the study of muscle lipid metabolism. NMR Biomed.

[25] Bhasin S, Calof OM, Storer TW, Lee ML, Mazer NA (2006). Drug insight: Testosterone and selective androgen receptor modulators as anabolic therapies for chronic illness and aging. Nat Clin Pract Endocrinol Metab.

[26] Sattler FR, Castaneda-Sceppa C, Binder EF, Schroeder ET, Wang Y (2009). Testosterone and growth hormone improve body composition and muscle performance in older men. J Clin Endocrinol Metab.

[27] Glintborg D, Christensen LL, Kvorning T, Larsen R, Brixen K (2013). Strength training and testosterone treatment have opposing effects on migration inhibitor factor levels in ageing men. Mediators Inflamm.

[28] Krssak M, Falk Petersen K, Dresner A, DiPietro L, Vogel SM (1999). Intramyocellular lipid concentrations are correlated with insulin sensitivity in humans: a 1H NMR spectroscopy study. Diabetologia.

[29] Perseghin G, Scifo P, De Cobelli F, Pagliato E, Battezzati A (1999). Intramyocellular triglyceride content is a determinant of in vivo insulin resistance in humans: a 1H-13C nuclear magnetic resonance spectroscopy assessment in offspring of type 2 diabetic parents. Diabetes.

[30] Dresner A, Laurent D, Marcucci M, Griffin ME, Dufour S (1999). Effects of free fatty acids on glucose transport and IRS-1-associated phosphatidylinositol 3-kinase activity. J Clin Invest.

[31] Pitteloud N, Mootha VK, Dwyer AA, Hardin M, Lee H (2005). Relationship between testosterone levels, insulin sensitivity, and mitochondrial function in men. Diabetes Care.

[32] Petersen KF, Befroy D, Dufour S, Dziura J, Ariyan C (2003). Mitochondrial dysfunction in the elderly: possible role in insulin resistance. Science.

[33] Allan CA, Strauss BJ, Burger HG, Forbes EA, McLachlan RI (2008). Testosterone therapy prevents gain in visceral adipose tissue and loss of skeletal muscle in nonobese aging men. J Clin Endocrinol Metab.

[34] Rebuffé-Scrive M, Mårin P, Björntorp P (1991). Effect of testosterone on abdominal adipose tissue in men. Int J Obes.

[35] Frederiksen L, Højlund K, Hougaard DM, Brixen K, Andersen M (2012). Testosterone therapy increased muscle mass and lipid oxidation in aging men. Age (Dordr).

[36] Bergman RN (2000). Non-esterified fatty acids and the liver: why is insulin secreted into the portal vein?. Diabetologia.

[37] Xu XF, De Pergola G, Björntorp P (1991). Testosterone increases lipolysis and the number of beta-adrenoceptors in male rat adipocytes. Endocrinology.

[38] Blouin K, Boivin A, Tchernof A (2008). Androgens and body fat distribution. J Steroid Biochem Mol Biol.

[39] Singh R, Artaza JN, Taylor WE, Gonzalez-Cadavid NF, Bhasin S (2003). Androgens stimulate myogenic differentiation and inhibit adipogenesis in C3H 10T1/2 pluripotent cells through an androgen receptor-mediated pathway. Endocrinology.

[40] Singh R, Artaza JN, Taylor WE, Braga M, Yuan X (2006). Testosterone inhibits adipogenic differentiation in 3T3-L1 cells: nuclear translocation of androgen receptor complex with betacatenin and T-cell factor 4 may bypass canonical Wnt signaling to down-regulate adipogenic transcription factors. Endocrinology.

[41] Snyder PJ, Peachey H, Hannoush P, Berlin JA, Loh L (1999). Effect of testosterone treatment on body composition and muscle strength in men over 65 years of age. J Clin Endocrinol Metab.

[42] Bhasin S, Pencina M, Jasuja GK, Travison TG, Coviello A (2011). Reference ranges for testosterone in men generated using liquid chromatography tandem mass spectrometry in a community-based sample of healthy nonobese young men in the Framingham Heart Study and applied to three geographically distinct cohorts. J Clin Endocrinol Metab.

[43] Wu FC, Tajar A, Beynon JM, Pye SR, Silman AJ (2010). Identification of late-onset hypogonadism in middle-aged and elderly men. N Engl J Med.

[44] Basu R, Dalla Man C, Campioni M, Basu A, Nair KS (2007). Effect of 2 years of testosterone replacement on insulin secretion, insulin action, glucose effectiveness, hepatic insulin clearance, and postprandial glucose turnover in elderly men. Diabetes Care.

[45] Nair KS, Rizza RA, O’Brien P, Dhatariya K, Short KR (2006). DHEA in elderly women and DHEA or testosterone in elderly men. N Engl J Med.

[46] Emmelot-Vonk MH, Verhaar HJ, Nakhai Pour HR, Aleman A, Lock TM (2008). Effect of testosterone supplementation on functional mobility, cognition, and other parameters in older men: a randomized controlled trial. JAMA.

[47] Svartberg J, Agledahl I, Figenschau Y, Sildnes T, Waterloo K (2008). Testosterone treatment in elderly men with subnormal testosterone levels improves body composition and BMD in the hip. Int J Impot Res.

[48] Mårin P, Holmäng S, Gustafsson C, Jönsson L, Kvist H (1993). Androgen treatment of abdominally obese men. Obes Res.

[49] Frederiksen L, Højlund K, Hougaard DM, Mosbech TH, Larsen R (2012). Testosterone therapy decreases subcutaneous fat and adiponectin in aging men. Eur J Endocrinol.

[50] Basaria S, Coviello AD, Travison TG, Storer TW, Farwell WR (2010). Adverse events associated with testosterone administration. N Engl J Med.

[51] Vigen R, O’Donnell CI, Barón AE, Grunwald GK, Maddox TM (2013). Association of testosterone therapy with mortality, myocardial infarction, and stroke in men with low testosterone levels. JAMA.

[52] Finkle WD, Greenland S, Ridgeway GK, Adams JL, Frasco MA (2014). Increased risk of non-fatal myocardial infarction following testosterone therapy prescription in men. PLoS One.

[53] Xu L, Freeman G, Cowling BJ, Schooling CM (2013). Testosterone therapy and cardiovascular events among men: a systematic review and meta-analysis of placebo-controlled randomized trials. BMC Med.

[54] Spitzer M, Huang G, Basaria S, Travison TG, Bhasin S (2013). Risks and benefits of testosterone therapy in older men. Nat Rev Endocrinol.

[55] Cappola AR (2013). Testosterone therapy and risk of cardiovascular disease in men. JAMA.

[56] Morales A (2014). Testosterone deficiency syndrome and cardiovascular health: Looking carefully at the evidence. Can Urol Assoc J.

